# First records of hydroid epibionts on the introduced macroalga *Gracilariaparvispora* in the Mexican Pacific

**DOI:** 10.3897/BDJ.12.e130248

**Published:** 2024-09-11

**Authors:** María A. Mendoza-Becerril, Paulina Murillo-Torres, Elisa Serviere-Zaragoza, Karla León-Cisneros, Alejandra Mazariegos-Villarreal, Juan Manuel López-Vivas, José Agüero

**Affiliations:** 1 El Colegio de la Frontera Sur (ECOSUR), Chetumal, Mexico El Colegio de la Frontera Sur (ECOSUR) Chetumal Mexico; 2 Departamento Académico de Ciencias Marinas y Costeras, Universidad Autónoma de Baja California Sur, La Paz, Mexico Departamento Académico de Ciencias Marinas y Costeras, Universidad Autónoma de Baja California Sur La Paz Mexico; 3 Centro de Investigaciones Biológicas del Noroeste (CIBNOR), La Paz, Mexico Centro de Investigaciones Biológicas del Noroeste (CIBNOR) La Paz Mexico; 4 Universidad Autónoma de Baja California Sur, La Paz, Mexico Universidad Autónoma de Baja California Sur La Paz Mexico; 5 Medusozoa México, La Paz, Mexico Medusozoa México La Paz Mexico

**Keywords:** Hydrozoa, interspecific relationship, La Paz Bay, Rhodophyta, symbiosis, taxonomy

## Abstract

**Background:**

The red macroalga *Gracilariaparvispora* is an introduced species in the Mexican Pacific. To date, there are no published studies on its sessile epibionts, including the hydrozoans and bryozoans, which are the dominant epibionts on macrophytes and of significant biological and economic interest.

**New information:**

This study provides insight into the faunal diversity of hydroids growing on *G.parvispora*. A total of 185 thalli from both herbarium specimens and field samples collected from five sites in La Paz Bay were revised. Each thallus size and the presence of hydroid epibionts in each thallus region were registered. Eight different hydrozoan taxa were growing on the red macroalgae, including the first recorded observation of *Obeliaoxydentata* in the Gulf of California. The sizes of the collected thalli were mostly under 7.0 cm, the maximum number of taxa per thallus was three and the thallus region containing the highest number of epibionts was in the middle. Significant differences were observed amongst the lengths of thalli with and without epibionts. The thalli with epibionts were larger than the thalli without epibionts. Similarly, significant differences were observed amongst the months. The pair-wise test revealed that each month exhibited distinctive epibiont taxa when compared to the others. This study highlights the lack of information on these associations. Further research is needed to understand whether introduced macroalgae can bring non-native epibiont species to an area.

## Introduction

Epibiosis is defined as an association between two or more living organisms, whereby one associate, the basibiont, provides a suitable surface for the settlement of the other(s), the epibionts (Wahl 1989, Wahl 2010). In marine ecosystems, macroalgae as basibionts provide a complex substrate by creating microhabitats where epibionts can attach, grow and reproduce (Schmidt and Scheibling 2006). Colonial invertebrates, commonly sessile epibionts of macroalgae, mostly belong to the phyla Cnidaria and Bryozoa, which present a planktonic larval phase that can adhere to a basibiont and form temporary or permanent colonies (Ryland 1962, Nishihira 1967, Stricker 1989, Connell 2000, Ryland 2005, Hiebert et al. 2020). The encrusting or erect colonies remain physically and physiologically connected through identical modular units, termed zooids in bryozoans and polyps in cnidarians (Mackie 1997, Hiebert et al. 2020). The epibiont cnidarians highlight the benthic polyps of class Hydrozoa, which are referred to as hydroids and these may have life cycles with a medusa phase, predominantly planktonic (Collins 2002, Oliveira et al. 2006, Cartwright and Nawrocki 2010), while the bryozoans have only benthic zooids (Bock 1982).

Several studies have observed hydrozoans and bryozoans growing on macroalgae (cf. Manríquez and Cancino 1996, Oliveira and Marques 2011, Cunha et al. 2017b, Carral-Murrieta et al. 2023) and have also highlighted the preference of some of these invertebrates for specific species and macroalgal morphologies (Gallardo et al. 2021, Carral-Murrieta et al. 2024). However, the ecological role and impact of introduced macroalgae as basibionts in coastal marine ecosystems are poorly understood. It has been found that introduced macroalgae-dominated systems have shown varying effects on local biodiversity and that epibiosis can be a mechanism to facilitate the invasion of epibionts as well (Arnold et al. 2015, Lazzeri and Auker 2022). Therefore, it is important to identify and inventorise the epibionts species growing on introduced macroalgae to monitor and manage the impact on local biodiversity. Additionally, macroalgae and colonial invertebrates are of biological, scientific and social interest due to their positive or negative economic impact on the pharmaceutical, food, biotechnology, fishing and aquaculture industries (cf. Grohmann (2008), Muñoz-Ochoa et al. (2010), Wood et al. (2012), Mouritsen (2013), Pereira (2018), Pinteus et al. (2018), Kintner and Brierley (2018), Ciavatta et al. (2020), Banagouro et al. (2022)).

Approximately 233 colonial invertebrate species have been recorded growing on marine macroalgae, of which 200 species are hydroids (Lippert et al. 2001, Oliveira and Marques 2007, Arnold et al. 2015, Gallardo et al. 2021, Carral-Murrieta et al. 2023). Some of these basibiont macroalgae are considered non-native or invasive macroalgae (Schaffelke et al. 2006, Davidson et al. 2015). However, the occurrence of hydrozoan epibionts has only been reported in nine species of introduced macroalgae. These comprise one green alga *Ulvalinza* Linnaeus, seven brown alga *Durvillaeaantarctica* (Chamisso) Hariot, Fucusdistichussubsp.evanescens (C.Agardh) H.T.Powell, *Himanthaliaelongata* (Linnaeus) S.F.Gray, *Macrocystispyrifera* (Linnaeus) C.Agardh, Sargassumhorneri (Turner) C.Agardh, *Sargassummuticum* (Yendo) Fensholt, *Undariapinnatifida* (Harvey) Suringar and one red macroalgae *Acanthophoraspicifera* (M.Vahl) Børgesen (Sarma 1974, Withers et al. 1975, Norton and Benson 1983, Kitching 1987, Migotto 1996, Sano et al. 2003, Wikström and Kautsky 2004, Oliveira and Marques 2007, Oliveira and Marques 2011, Kuhlenkamp and Kind 2013, Arnold et al. 2015, Gutow et al. 2015, Kim et al. 2019, Avila et al. 2020, Carral-Murrieta et al. 2023, Mendoza-Becerril et al. 2023, Carral-Murrieta et al. 2024). Still, only some of these studies address interdependent distribution patterns, dispersal and interaction with local, non-indigenous or invasive species (e.g. Kuhlenkamp and Kind (2013), Kim et al. (2019), Avila et al. (2020)).

In Mexico, records of non-native or invasive macroalgae range from seven (Okolodkov et al. 2007) to 15 species (Aguilar-Rosas et al. 2014), all of them reported along the Pacific coast and only *A.spicifera* has been reported as a non-native alga for Pacific and Atlantic coasts (Mendoza-Becerril et al. 2023). For three species (*A.spicifera*, *Sargassumhorneri* and *S.muticum*), their sessile epibionts are known (Mendoza-Becerril et al. 2023, Carral-Murrieta et al. 2024), but not in the others, such as the rhodophyte *Gracilariaparvispora* I.A.Abbott (Gracilariaceae), which has been mentioned as an invasive species in the Baja California Peninsula, based on morphological, anatomical and molecular data (García-Rodríguez et al. 2013). Currently, it is also recorded in the States of Oaxaca, Chiapas, Guerrero, Colima and Sinaloa (Dreckmann 1999, García-Rodríguez et al. 2013, Orduña-Rojas et al. 2013, Dreckmann and Sentíes 2014, Krueger-Hadfield et al. 2016, Acosta-Calderón and Chávez-Sánchez 2019, Méndez-Trejo et al. 2021).

*Gracilariaparvispora*, also known as limu ogo or long ogo, was described from Kaneohe Bay, Oahu, Hawaiian Islands, USA (Abbott 1985, Lembi and Waaland 1988) and, since then, it has been recorded in Korea, Japan and China (Kim et al. 2008, Guiry and Guiry 2024). However, the origin of *G.parvispora* in Hawaii is unclear (Abbott 1999, Nelson et al. 2009) and its epibionts are also unknown. It is one of the three most sought-after seaweeds for food in the Hawaiian Islands and a potential source of agar (Abbott 1999, Krueger-Hadfield et al. 2016).

In this context, the present study aimed to analyse the hydrozoans associated with an introduced macroalgae, *G.parvispora* and determine potential assemblages of these epibionts according to the macroalga's morphological characteristics, based on an analysis of herbarium and collected thalli in a subtropical bay of the Gulf of California, Baja California Sur (BCS).

## Materials and methods

### Herbarium specimens

**Figure 1.**
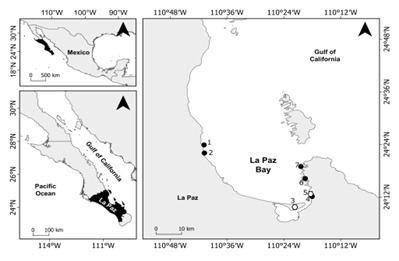
Herbarium material (white hexagon) and sampling sites (black points). 1) Port of San Juan de la Costa, 2) ROFOMEX SJC, 3) El Mogote, 4) La Concha, 5) El Caimancito, 6) UABCS Pichilingue, 7) Punta Diablo.

*Gracilariaparvispora* dried specimens were obtained from the Phycological Herbarium of the Autonomous University of Baja California Sur (FBCS) and were collected in La Paz Bay: El Mogote, La Concha and El Caimancito, which are beaches frequented by tourists (Table 1, Fig. 1, Suppl. material 1).

### Field samples

Based on previous reports of *G.parvispora* in La Paz Bay, BCS, five sites were visited, including disturbed and undisturbed environments. The port of San Juan de la Costa, the Roca Fosfórica Mexicana phosphorite mine at San Juan de la Costa (ROFOMEX SJC), La Concha, the port of the Autonomous University of Baja California Sur Pichilingue (UABCS Pichilingue) and Punta Diablo (Table 1, Figure 1, Suppl. material 1). In Punta Diablo, natural substrates were present without direct contact with nautical traffic. The other four sites had various artificial substrates and were exposed to nautical traffic or anthropogenic activities. ROFOMEX SJC and the port of San Juan de la Costa are adjacent to a phosphorite mine with a daily production of 6,000 tonnes (Servicio Geológico Mexicano 2018). Its port is located 2.8 km north of the fiscal dock in La Paz Bay. La Concha is a tourist beach with hotel activities and a recreational diving company. UABCS Pichilingue is located 3.2 km north of the Pichilingue’s port, where tourist and commercial ships arrive (Fig. 1).

**Figure 2.**
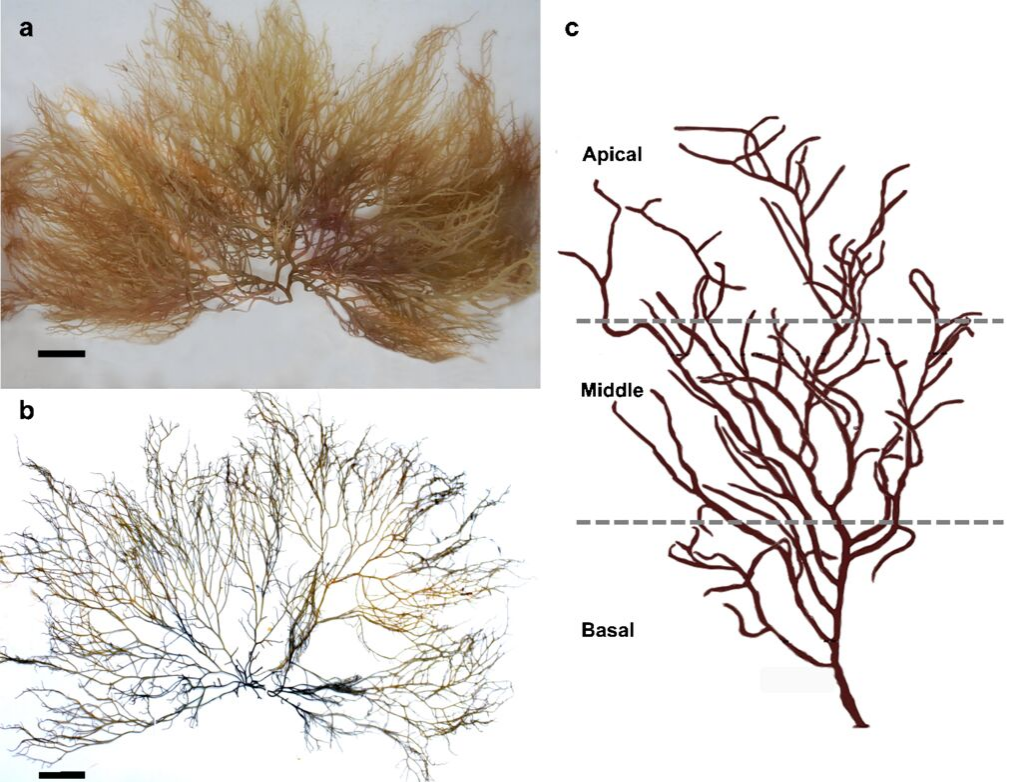
*Gracilariaparvispora*. **a** Field sample of La Concha, scale equals 2.0 cm; **b** Herbarium specimen under code FBCS2490, El Caimancito, scale equals 2.0 cm; **c** Scheme with regions for recording epibionts.

Following periodic changes in surface temperature and the entrance and retirement of tropical waters in La Paz Bay and Gulf of California (Santamaría‐del‐Ángel et al. 1994, Flores-Ramírez et al. 1996, Guevara-Guillén et al. 2015), the sites were visited in spring (transition period between cold and warm waters), winter (cold waters) and summer (warm waters) during the annual cycle 2021–2022. The complete macroalgal thalli were randomly sampled manually by the same team and using a knife or scraping artificial or natural substrates (n ≥ 10) by snorkelling and scuba diving in sites with depths of more than three metres. The samples were fixed in 96% ethanol for morphological analysis. The depth (m) was measured *in situ*, at the snorkelling sites, the depth was measured with a sounding weight and calibrated line marked from 0 to 5 m in 20 cm increments, while at the dive sites, it was measured with a dive computer. Thalli were identified according to morphology (Fig. 2) following Abbott (1985), Dreckmann (1999) and García-Rodríguez et al. (2013) descriptions. In the laboratory, the length (cm) and presence of cystocarps of herbaria and collected thalli of *G.parvispora* were registered.

Each thallus was divided into three equal regions (basal, middle and apical) and the presence or absence of hydrozoans on the thallus was recorded. The basal region consisted of the first third closest to the disc and part of the stem, the middle region included the central part of the alga and the last third of the thallus from the middle part to the tips of the alga was catalogued as the apical region (Fig. 2).

Epibionts were identified with the support of taxonomic descriptions and compilations available in literature (e.g. Millard (1975), Mendoza-Becerril et al. (2020)) and the nomenclature used was based on a study by Maronna et al. (2016) for Leptothecata hydroids and the World Register of Marine Species (WoRMS Editorial Board 2024) for other hydroids. After the analysis, the specimens (algae and invertebrates) were deposited in the Macroalgae Laboratory from Centro de Investigaciones Biológicas del Noroeste, S.C.

For each taxon, we provided the material section (locality, depth, data, habitat, data generalisations), diagnosis and notes [figure, type locality, references for a detailed description of the species, taxonomic status with a unique and persistent identifier that assures the taxonomic quality control denominated “AphiaID” (Vandepitte et al. 2015) and remarks (only for taxa with additional information)]. In the material examined, we included the sampling site and date, temperature (°C), salinity (PSU), depth (m) and presence of epibionts in the specific macroalgal regions. Descriptions, taxonomic status and dichotomous key are included only for specimens identified to species level.

The significant differences in size between thalli with and without epibionts were tested by one-way permutational multivariate ANOVA (PERMANOVA) using untransformed data and Euclidean distances. Additionally, PERMANOVA was used to determine whether epibiont assemblages differed significantly amongst: 1) month, 2) cystocarp and non-cystocarp thalli and 3) basal, middle and apical thallus regions. The presence-absence data matrix was analysed using the Jaccard similarity measure with 9999 permutations and significance was set at p < 0.05. When a significant effect was found, post hoc paired comparisons between factor levels were performed (Anderson 2001, McArdle and Anderson 2001). Percentage similarity analysis (SIMPER) was also used to determine the contribution of species to within-group similarity and between-group dissimilarity (Clarke et al. 2014). Statistical analysis was performed in PRIMER v.6 using the PERMANOVA+ add-on software (Clarke and Gorley 2006, Anderson et al. 2008).

## Data resources

### Gracilariaparvispora and hydrozoan epibionts

**Figure 3.**
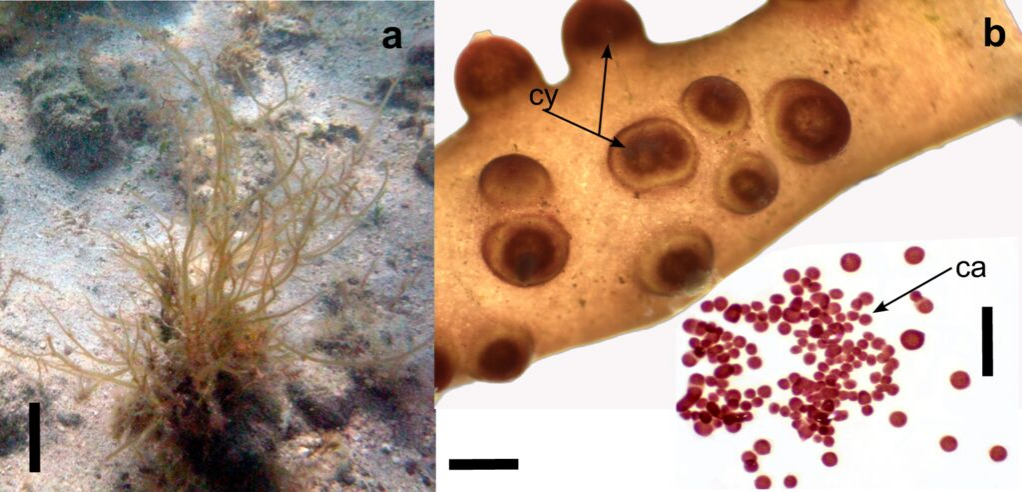
*Gracilariaparvispora*. **a** macroalgae on rock with yellow colouration, La Concha, scale equals 5.0 cm; **b** cystocarps (cy), scale equals 5.0 mm and carpospores (ca), scale equals 0.1 mm.

Thalli of *G.parvispora* were mainly found in sandy substrates and can be on pebbles, rocky and bivalve shells (Fig. 3a). The thallus is cylindrical, except in the branches, which are flattened to cylindrical, usually with three orders of branches, from 0.5 to 4.0 mm in diameter and present irregular dichotomous branches, sympodial without a defined main axis. Thallus has a flaccid consistency and its colouration can vary: yellow, green, red or brown. There are large medullary cells from 90 to 280 mm in diameter, with two cortical cells surrounding the medullary cells. Cystocarps are present from 2.0 to 5.0 mm in diameter (Fig. 3b), as well as chain carpospores of ovoid to the slightly spherical shape from 15 to 35 μm in diameter (Fig. 3b).

**Figure 4.**
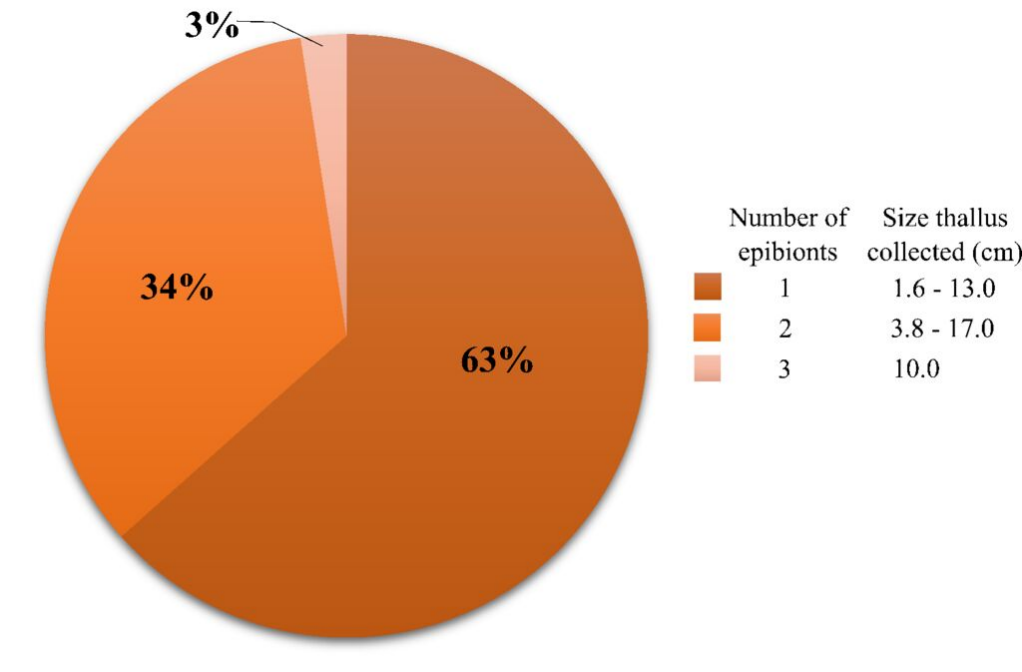
Percentage and size (cm) of *Gracilariaparvispora* thalli with the number of epibiont taxa found.

The total number of thalli analysed was 185, of which 10 were from herbarium specimens. The length ranged from 1.4 to 36 cm, with 88% of the samples under 7.0 cm. Eight percent presented cystocarps (15 thalli) and 22% presented hydrozoan epibionts (41 thalli), which were found in thalli sizes from 1.6 to 17.0 cm and a maximum of three taxa per thallus were recorded. Sixty-three percent of the thalli with epibionts had two species of epibiont hydroids (Fig. 4).

**Figure 5.**
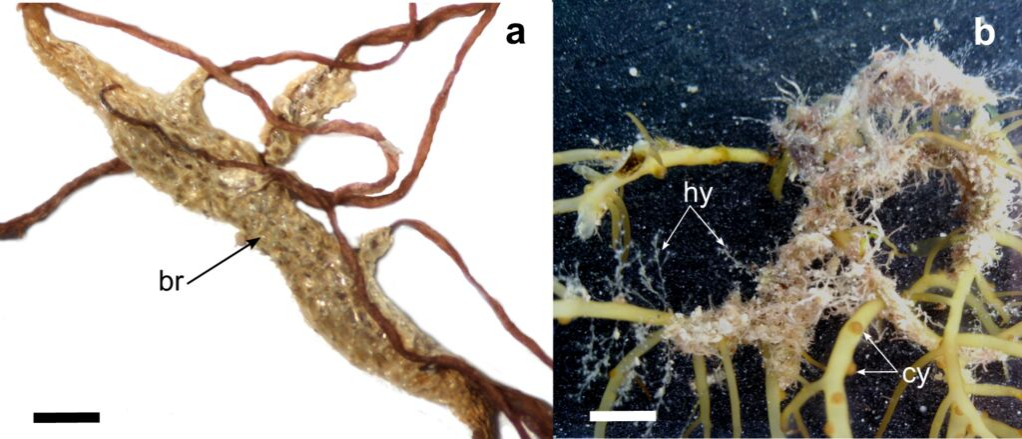
*Gracilariaparvispora* with colonial epibionts. **a** Bryozoa epibionts, scale equals 1.0 mm; **b** Hydrozoa epibionts, scale equals 5.0 mm. Abbreviations: br, Bryozoa epibiont; cy, cystocarps; hy, Hydrozoan epibionts.

Six species and two genera of the Hydrozoa epibionts were identified and were observed only in the collected thalli. In addition, no bryozoans were found in the collected thalli, but some belonging to the Gymnolaemata class, order Cheilostomatida, were observed on four herbarium thalli (Fig. 5). It should be noted that the bryozoans observed were morphologically damaged and it was not possible to identify them at a lower level of order. As this was the first record of epibionts in this macroalga, the epibiont hydroids were integrated into a dichotomous key and taxonomically described.

## Checklists

### Taxonomy and morphological descriptions of Hydrozoa epibionts

#### Hydroidolina

Collins, 2000

EDD7CE2C-EC22-5F44-963F-F78FB75AABEA

#### “Anthoathecata”

Cornelius, 1992

B9983DD8-F22B-5D11-8CA3-4AF6CE451DA1

#### "Filifera"

Kühn, 1913

5BF728CD-445E-50E1-89DC-2C2ABF85E7D8

#### Oceaniidae

Eschscholtz, 1829

9DF2995B-5F88-5C88-96E6-EE1EB6A21808

#### Corydendrium

Van Beneden, 1844

C6B210E7-D429-5701-A778-1F90FC4B2F18

#### Corydendrium sp.

F1C2C083-5E50-5473-B361-262FFFBB0B2E

##### Materials

- **Type status:** Other material. **Location:** locality: La Concha; minimumDepthInMeters: 0.5; maximumDepthInMeters: 1.0; **Event:** year: 2022; month: 02; day: 11; habitat: middle and apical macroalgae regions; **Record Level:** dataGeneralizations: 18°C; 36 PSU

##### Notes

Fig. 6a

Detailed description in Calder (1988).

##### Diagnosis

Colony erect, hydrocaulus polysiphonic, irregularly branched; branches partly adnate to hydrocaulus. Exosqueleton thick with detritus, becoming thin at hydranth base and terminating below filiform tentacles; tentacles scattered over hydranth. Without gonophores.

#### Leptothecata

Cornelius, 1992

5E253AB1-2D4D-5AD4-B580-B3983A1ABC7D

#### Macrocolonia

Leclère, Schuchert, Cruaud, Couloux and Manuel 2009

1752D65C-CB7D-533F-927F-6580DB645036

#### Plumupheniida

Maronna, Miranda, Peña Cantero, Barbeitos and Marques 2016

3479E2B3-F951-53CA-A2C6-96A28ECB51B9

#### Plumulariida

Bouillon, 1984

2F22A6FB-EAA9-594D-B9BD-EEBD2E72AC61

#### Kirchenpaueriidae

Stechow, 1921

58C70B74-48AC-51E7-BB24-296261C04CEB

#### Ventromma

Stechow, 1923

F9425B4C-43E6-559A-8F50-4CC50C128B1F

#### Ventromma halecioides

(Alder, 1859)

EC35AE04-CCC7-504C-904C-34A5ABE9CEA5

##### Materials

- **Type status:** Other material. **Location:** locality: La Concha; minimumDepthInMeters: 0.5; maximumDepthInMeters: 1.0; **Event:** year: 2021; month: 6; day: 19; habitat: all macroalgae regions; **Record Level:** dataGeneralizations: 18°C; 36 PSU
- **Type status:** Other material. **Location:** locality: La Concha; minimumDepthInMeters: 0.5; maximumDepthInMeters: 1.0; **Event:** year: 2022; month: 2; day: 11; habitat: all macroalgae regions; **Record Level:** dataGeneralizations: 18°C; 36 PSU

##### Notes

Fig. 6b

Type locality. Cullercoats and Roker, England (Alder 1859).

Detailed description in Calder (1997), Peña-Cantero and García-Carrascosa (2002), Mendoza-Becerril et al. (2020).

**Figure 6.**
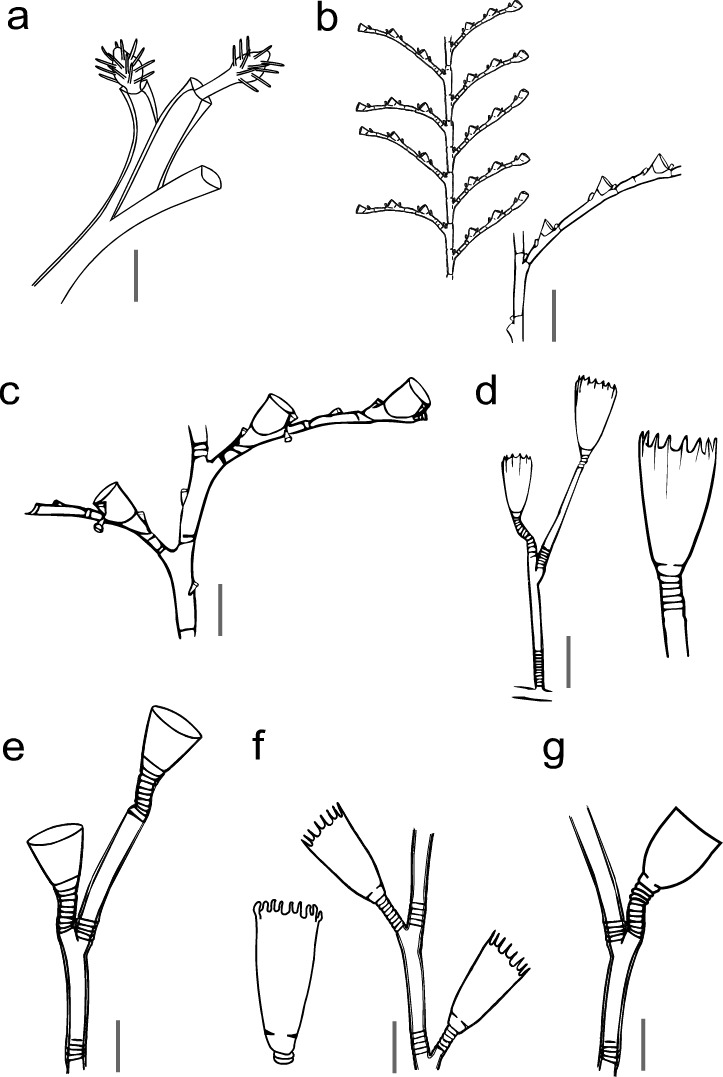
Hydrozoan epibionts. **a** *Corydendrium* sp.: part of hydrocaulus with two hydranths, scale equals 0.5 mm; **b** *Ventrommahalecioides*: part of a hydrocaulus with the proximal end of a hydrocladium, hydrothecae, nematothecae, scale equals 0.3 mm; **c** *Plumulariafloridana*: part of a hydrocaulus with the proximal end of a hydrocladium, hydrothecae and nematothecae, scale equals 0.1 mm; **d** *Clytialinearis*: part of hydrocaulus with hydrothecae, scale equals 0.8 mm; **e** Obeliacf.dichotoma: part of hydrocaulus with hydrothecae, scale equals 0.2 mm; **f** *Obeliaoxydentata*: part of hydrocaulus with hydrothecae, scale equals 0.3 mm; **g** *Obeliatenuis*: part of hydrocaulus with a hydrotheca, scale equals 0.1 mm.

Taxonomic status. Unaccepted (see Fig. 6b remarks). AphiaID 117678.

**Remarks**. Recent molecular studies support the validity of species with sufficient genetic divergence from Kirchenpaueria, forming a sister clade to the rest of the family Kirchenpaueriidae (Peña-Cantero et al. 2010, Maronna et al. 2016, Moura et al. 2018). Therefore, we follow this genetic evidence and the presence of bithalamic nematothecae (c.f. Calder 1997), we consider our specimens to belong to *V.halecioides*.

##### Diagnosis

Colony erect, with creeping hydrorhiza. Hydrocaulus branched, monosiphonic, divided at regular intervals into internodes, each with one distal nematotheca and one hydrocladial apophysis. Exoskeleton with a visible layer corresponding to perisarc. Hydrocladia alternate, unbranched, with up to four thecate internodes. Thecate internodes with a distal hydrotheca, a median inferior nematotheca and a median superior nematothecae. Hydrotheca cup-shaped with margin entire. Without gonothecae.

#### Plumulariidae

McCrady, 1859

93EEFF64-DB15-50B7-9A5A-D812A8773B2A

#### Plumularia

Lamarck, 1816

A352577E-6490-56F0-BED5-0EA2EDDCCDB4

#### Plumularia floridana

Nutting, 1900

88061294-77A6-5A85-B6F2-375520403625

##### Materials

- **Type status:** Other material. **Location:** locality: La Concha; minimumDepthInMeters: 0.5; maximumDepthInMeters: 1.0; **Event:** year: 2022; month: 2; day: 11; habitat: middle macroalgae regions; **Record Level:** dataGeneralizations: 18°C; 36 PSU

##### Notes

Fig. 6c

Type locality. USA, two miles west of Cape Romano, Florida (Nutting 1900).

Detailed description in Calder (1983), Calder (1997), Mendoza-Becerril et al. (2020).

Taxonomic status. Accepted. AphiaID 117821.

##### Diagnosis

Colonies erect arising from creeping hydrorhiza. Hydrocaulus monosiphonic, branched; medium and distal part of the hydrocaulus distinctly divided into regular internodes by transverse nodes; internodes straight, but slightly curved distally; each internode with a distal apophysis and with three nematothecae, two axillary and one median opposite to apophysis. Hydrocladia alternate, unbranched, with alternate athecate and thecate internodes. Nematothecae conical, bithalamic and movable. Hydrotheca cup-shaped; margin entire, without intrathecal septum. Without gonothecae.

#### Statocysta

Leclère, Schuchert, Cruaud, Couloux and Manuel 2009

B2B8E8A9-0F74-554A-B623-05F623AE3A4D

#### Proboscoida

Broch, 1910

897E766A-9B37-59BF-8533-0DE8B994A18B

#### Obeliida

Maronna, Miranda, Peña Cantero, Barbeitos and Marques, 2016

0D079FEA-062A-5806-B565-86B0E289CAAB

#### Clytiidae

Cockerell, 1911

75F23D4D-0185-54A2-92C8-76C2574341DA

#### Clytia

Lamouroux, 1812

9239C421-6F0B-59D8-8FE6-3448D92634F7

#### Clytia linearis

(Thornely, 1900)

39A4C305-BCAE-517A-A720-E9AE25F1EF07

##### Materials

- **Type status:** Other material. **Location:** locality: La Concha; minimumDepthInMeters: 0.5; maximumDepthInMeters: 1.0; **Event:** year: 2021; month: 6; day: 19; habitat: middle macroalgae regions; **Record Level:** dataGeneralizations: 18°C; 36 PSU
- **Type status:** Other material. **Location:** locality: La Concha; minimumDepthInMeters: 0.5; maximumDepthInMeters: 1.0; **Event:** year: 2022; month: 2; day: 11; habitat: middle macroalgae regions; **Record Level:** dataGeneralizations: 18°C; 36 PSU

##### Notes

Fig. 6d

Type locality. Papua New Guinea: Blanche Bay, New Britain (Thornely 1900).

Detailed description in Calder (1991b), Mendoza-Becerril et al. (2020).

Taxonomic status. Accepted. AphiaID 117370.

##### Diagnosis

Colonies erect, sympodial, occasionally branching. Erect stems monosiphonic, arising from a creeping hydrorhiza. Exoskeleton thin. Internodes with annulations at the base and upward curved apophysis, adjacent to hydrothecal pedicel; pedicel with distal hydrotheca and annulations along its whole length. Hydrotheca cylindrical, with a diaphragm thin, transverse, hydrothecal margin with triangular cusps and pleats originating at apex of each cusp and continuing downwards to upper part of hydrothecal wall. Without gonothecae.

#### Clytia sp.

1CFD7458-5F64-5D61-8534-5B10C1997A5B

##### Materials

- **Type status:** Other material. **Location:** locality: La Concha; minimumDepthInMeters: 0.5; maximumDepthInMeters: 1.0; **Event:** year: 2022; month: 2; day: 11; habitat: middle macroalgae regions; **Record Level:** dataGeneralizations: 18°C; 36 PSU

##### Notes

Detailed description in Calder (1991b).

##### Diagnosis

Stolonal colony. Pedicel annulated basally and distally. Hydrothecae campanulate with distinct cusps; true diaphragm present; without spherule. Gonothecae absent.

#### Obeliidae

Haeckel, 1879 Genus Obelia Péron and Lesueur, 1810

6F8357AB-0934-5D2C-81C7-F3B3C98A9EAF

#### Obelia

Péron & Lesueur, 1810

3581F143-1032-5C43-9F4A-775847306720

#### Obelia cf. dichotoma

(Linnaeus, 1758)

4517DE27-CE63-5E17-8C32-C70960B39193

##### Materials

- **Type status:** Other material. **Location:** locality: La Concha; minimumDepthInMeters: 0.5; maximumDepthInMeters: 1; **Event:** year: 2021; month: 6; day: 19; habitat: apical macroalgae regions; **Record Level:** dataGeneralizations: 18°C; 36 PSU
- **Type status:** Other material. **Location:** locality: UABCS Pichilingue; minimumDepthInMeters: 0.0; maximumDepthInMeters: 1; **Event:** year: 2021; month: 7; day: 15; habitat: basal macroalgae regions; **Record Level:** dataGeneralizations: 28°C; 36 PSU
- **Type status:** Other material. **Location:** locality: La Concha; minimumDepthInMeters: 0.5; maximumDepthInMeters: 1; **Event:** year: 2022; month: 2; day: 11; habitat: all macroalgae regions; **Record Level:** dataGeneralizations: 18°C; 36 PSU

##### Notes

Fig. 6e

Type locality. Southwest England (Cornelius 1975).

Detailed description in Mendoza-Becerril et al. (2020).

Taxonomic status. Accepted. AphiaID 117386.

Remarks. It is now widely accepted and supported that the traditional concept of O.cf.dichotoma (cf. Cornelius (1995)) comprises multiple cryptic lineages (Calder 2013, Calder et al. 2014, Cunha et al. 2017a, Calder et al. 2019, Cunha et al. 2020) and in the eastern Pacific, affinities still need to be determined between local populations (Calder et al. 2019), mainly because their lineages are not distinguished from each other by morphometric analyses (Cunha et al. 2020). Therefore, molecular studies will be necessary to delimit the eastern Pacific lineages.

##### Diagnosis

Colonies erect, sympodial, in some cases with first-order branches. Exoskeleton thin. Stem monosiphonic, divided into internodes at regular intervals. Internodes with annulations at the base and one distal apophysis alternately given off the hydrothecal pedicel. Hydrotheca short and conical, with diaphragm oblique and margin entire. Hydrothecal pedicel with annulations along its whole length. With conical gonothecae with a short distal neck, arising from the base of the hydrothecal pedicel or the axis of the main stem and branches.

#### Obelia oxydentata

Stechow, 1914

AEC898C7-BC71-5EA0-B0D9-1AE46C50F28D

##### Materials

- **Type status:** Other material. **Location:** locality: UABCS Pichilingue; minimumDepthInMeters: 0.0; maximumDepthInMeters: 1.0; **Event:** year: 2021; month: 7; day: 15; habitat: middle and apical macroalgae regions; **Record Level:** dataGeneralizations: 28°C; 36 PSU

##### Notes

Fig. 6f, Fig. 7

Type locality. United States: Virgin Islands, St. Thomas, Charlotte Amalie (Stechow 1914).

Detailed description in Stechow (1914).

Taxonomic status. Accepted. AphiaID 766210.

**Remarks.** Colony morphology and size support evidence that this species differs from *Obeliabidentata* Clark, 1875. Previous studies have discussed the reason for the recognition of the species, supporting the correct identification of the species (Calder 2013, Calder 2019, Calder et al. 2019). This species is 1–60 mm tall, with predominant sizes from 1 to 10 mm (Calder et al. 2019, Calder 2020, Calder et al. 2021). The species has been recorded in other localities of the Pacific: Coconut Island Reef, Hawaii; Salinas Yacht Club, Ecuador (Calder 2020, Calder et al. 2021); and Oaxaca, Mexico (Ramos-Morales et al. 2024). However, in this last record, the number of cusps (7-10 vs. 15-20) differs from the original description and further descriptions (cf. Stechow (1914), Calder et al. (2019), Calder (2020), Calder et al. (2021), Ramos-Morales et al. (2024), this study) and, in fig. 3g of Ramos-Morales et al. (2024), the cuspids are unclear.

##### Diagnosis

Colonies monosiphonic, erect from 1-4 mm. Exoskeleton thin. Hydrothecal pedicel unbranched with a single hydrotheca at the distal end. Pedicel with 4-6 annulations (0.1-0.05 mm length); branches arising from curved and short lateral apophysis. Hydrothecae (0.2 mm wide) straight to the slightly oblique diaphragm and bi-mucronate marginal cusps (16-20 in total). Cusps are slightly rounded, with deep, rounded spaces between each other, alternately differing slightly in depth. Without gonophores.

#### Obelia tenuis

Fraser, 1938

AD0C40B5-EF7D-5989-83D2-09B5223FB62A

##### Materials

- **Type status:** Other material. **Location:** locality: La Concha; minimumDepthInMeters: 0.5; maximumDepthInMeters: 1.0; **Event:** year: 2022; month: 2; day: 11; habitat: middle macroalgae regions; **Record Level:** dataGeneralizations: 18°C; 36 PSU

##### Notes

Fig. 6g

Type locality. Ecuador: Santa Elena Bay (Calder et al. 2009).

Detailed description in Mendoza-Becerril et al. (2020).

Taxonomic status. Accepted. AphiaID 832333.

**Remarks.** Recent morphological studies support the validity of species with sufficient support. Therefore, we follow the morphological evidence indicated in Mendoza-Becerril et al. (2020).

##### Diagnosis

Colonies erect, sympodial and branching. Stem monosiphonic, divided into nodes and internodes. Internodes with annulations at the base and distal apophysis alternately given off the hydrothecal pedicel or branches. Hydrothecal pedicels are short, with annulations throughout. Hydrothecae with a margin slightly waived or with fine longitudinal folds. Hydrothecal diaphragm straight to slightly oblique. Without gonothecae.

## Identification Keys

### Dichotomous key of Hydrozoa epibionts of *Gracilariaparvispora*

**Table:** 

1	Hydroids without hydrotheca or with a firm pseudohydrotheca and detritus enveloping the hydroid.	2
–	Hydroids with hydrothecae.	4
2	Hydroids with at least a few capitate tentacles.	3
–	Hydroids with filiform tentacles only.	Filifera
3	Tentacles scattered around the hydrant, not arranged in distinct whorls. Gonophores in the form of fixed sporosacs.	*Corydendrium*
–	Hydrants with tentacles arranged in circle(s) at distal end.	Other Filifera
4	Hydrotheca adnate to hydrocaulus, nematothecae are present.	5
–	Hydrotheca not adnate to hydrocaulus, nematothecae are absent.	7
5	Hydrotheca without lateral nematothecae.	*Ventrommahalecioides*
–	Hydrotheca with lateral nematothecae.	6
6	Hydrocladia with more than one hydrotheca; hydrotheca adnate to internode; abcaulinar wall of hydrotheca straight, abcaulinar wall of hydrotheca straight.	*Plumulariafloridana*
–	With one hydrotheca per hydrocladia, hydrotheca partially adnate to internode; abcauline wall of hydrotheca slightly curved abcaulinar wall of the hydrotheca slightly curved.	*Monotheca*
7	Stolonal colony, with subhydrotecal spherule; hydrotheca with or without diaphragm.	others Proboscoida
–	Erect or stolonal colony, without a subhydrotecal spherule; hydrotheca with diaphragm.	8
8	Hydrotheca with cusps, cylindrical, with a diaphragm thin and transverse.	9
–	Hydrotheca margin without cusps.	10
9	Hydrothecal margin with about 11 to 16 cusps, deeply cut teeth separated by U-shaped incisions; margin scalloped in cross-section, with V-shaped pleats extending inwards towards hydrothecal cavity; each pleat originating at the apex of each tooth and continuing downwards to the upper part of hydrothecal wall.	*Clytialinearis*
–	Hydrothecal margin with about 15 - 20 long cusps, slightly rounded, with deep, rounded spaces between them, which alternately show a slight difference in depth so that an indistinct paired arrangement occurs.Hydrothecal margin with about 15 - 20 long cusps, slightly rounded, with deep, rounded spaces between them, which alternately show a slight difference in depth so that an indistinct paired arrangement occurs.	*Obeliaoxydentata*
10	Hydrothecal diaphragm oblique. Hydrothecal pedicel with annulations along its whole length.	Obeliacf.dichotoma
–	Hydrothecae with straight margin.	*Obeliatenuis*

## Analysis

**Figure 8.**
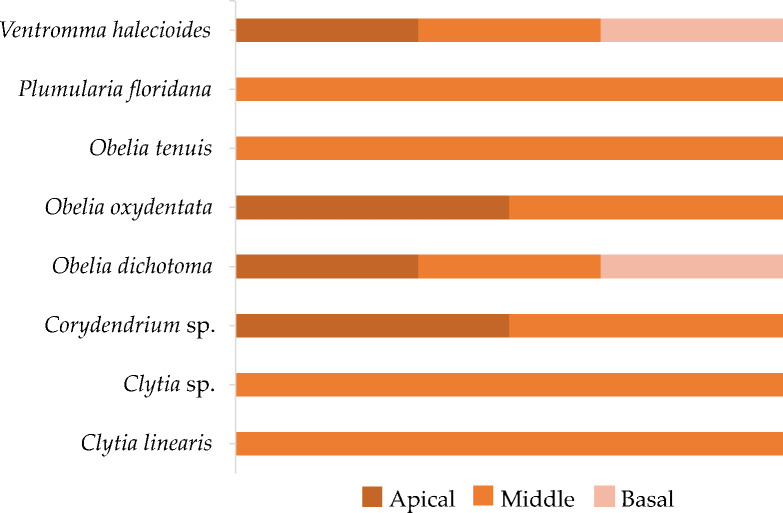
Hydrozoan epibiont taxa by thalli region of *Gracilariaparvispora* from La Paz Bay, Baja California Sur, Mexico.

Most of the epibiont taxa were recorded in the middle of the thalli, the same region that only the hydrozoans *Clytia* sp., *C.linearis*, *O.tenuis* and *P.floridana* were observed. Significant differences were observed amongst the lengths of thalli with and without epibionts (Pseudo-F = 3.04, p(perm) < 0.01, gl = 1, 118). The thalli with epibionts were larger than the thalli without epibionts (7.07 ± 2.89 cm and 4.89 ± 2.61 cm, respectively). Likewise, significant differences were observed amongst the months (Pseudo-F = 9.60, p(perm) < 0.01, gl = 2, 117), the pair-wise test revealing that each month exhibited distinctive epibiont taxa when compared to the others (p(perm) < 0.01). However, there was no significant difference between thalli with and without cystocarps (Pseudo-F = 2.35, p(perm) = 0.05, gl = 1, 118) and region (Pseudo-F = 0.39, p(perm) = 0.871, gl = 2, 143). SIMPER analysis revealed that the species that contributed the most to similarity within groups and dissimilarity between groups were *O.dichotoma*, *V.halecioides* and *C.linearis* (Table 2, Fig. 8).

## Discussion

A total of eight hydrozoan epibionts species were recorded for the first time in *G.parvispora* thalli. One of the most notable differences in the presence of epibionts was the quantity found in field and herbarium thalli. The latter yielded fewer epibionts, possibly due to the preparation of the thalli before being fixed, as these were rinsed free of any sand or debris, without emphasis on the conservation of the epibiont fauna. Therefore, epibionts with erect growth and calcareous or chitinous exoskeletons are only sometimes preserved when dried since they become brittle and are often lost in herbarium samples (M.A.M.-B. and K.L.-C. pers. obs.); for example, calcareous bryozoans were observed in the herbarium thalli. In the collected thalli, no other encrusting epibionts were observed, such as bryozoans or sponges, even though literature shows evidence that these epibionts co-exist (cf. Maggioni et al. (2020)) and are considered dominant epibionts (Altuna 1994, Gappa and Sabattini 2007) due to their ability to survive the spatial competition. This is a primordial characteristic in colonial organisms since they adapt under selective pressure to environmental changes (Jackson 1977, Gili et al. 2000).

All species of hydrozoan epibionts had already been recorded in La Paz Bay and the Mexican Pacific (cf. Estrada-González et al. (2023a)), except *Obeliaoxydentata*, whose records were restricted to the Galápagos Islands on bryozoan *Amathiaverticillata* (delle Chiaje, 1822) (Calder et al. 2019), Ecuador mainland on *Pennariadisticha* Goldfuss, 1820 and another hydroid stem (Calder et al. 2021) and the southern Mexican Pacific on PVC plates (Ramos-Morales et al. 2024). Therefore, this study's record is the first observation in La Paz Bay and the Gulf of California. Of the taxa recorded, only O.cf.dichotoma is considered invasive and *O.oxydentata* introduced in Mexican waters (CONABIO 2015, Ramos-Morales et al. 2024). Genetic analyses are suggested to resolve cryptic lineages and help explain many of the geographic and ecological patterns of hydroids recorded. The effects of this epibiosis are largely unknown and, therefore, we believe that it is important to monitor these introduced species and determine its level of invasiveness on marine ecosystems.

The epibiont hydroids of *G.parvispora* represent 22% of the current hydroids diversity of La Paz Bay (cf. Mendoza-Becerril et al. (2020), Mendoza-Becerril et al. (2022), Estrada-González et al. (2023a), Estrada-González et al. (2023b)). The hydroids species in La Paz Bay have been recorded in natural and artificial substrates, with macroalgae being their main settlement substrate (89%). Four of them were generalist: *C.linearis* recorded on 14 substrates (artificial substrate, Ascidian, Bryozoa, coral, Crustacea, detritus/sand, Hydrozoa, macroalgae, Mollusca, Polychaeta, Porifera, rock, unknown substrate, wood), O.cf.dichotoma recorded on 11 substrates (artificial substrate, Ascidian, Bryozoa, coral, Crustacea, Hydrozoa, macroalgae, Polychaeta, Porifera, rock, unknown substrate), *V.halecioides* recorded on 10 substrates (Bryozoa, calcareous organisms unidentified, coral, Crustacea, Hydrozoa, macroalgae, Polychaeta, Porifera, rock, unknown substrate) and *P.floridana* on nine substrates (artificial substrate, Ascidian, Bryozoa, calcareous organisms unidentified, Crustacea, macroalgae, Porifera, rock, unknown substrate) (Mendoza-Becerril et al. 2020, Mendoza-Becerril et al. 2022, Estrada-González et al. 2023a, Estrada-González et al. 2023b and this study). The most frequent species, O.cf.dichotoma and *V.halecioides*, correspond to erect branched colonies and substrate generalists (Calder 1991a). Additionally, these species are commonly distributed in La Paz Bay and as epibionts of macroalgae worldwide; for example, O.cf.dichotoma is an abundant species on *Sargassum* spp., while *V.halecioides* is dominant on *Cystoseira* spp. (Faucci and Boero 2000, Estrada-González et al. 2023a, Carral-Murrieta et al. 2024).

Globally, 31 species of epibiont hydroids are reported growing on non-native or invasive macroalgae (Sarma 1974, Withers et al. 1975, Norton and Benson 1983, Kitching 1987, Migotto 1996, Sano et al. 2003, Wikström and Kautsky 2004, Oliveira and Marques 2007, Oliveira and Marques 2011, Kuhlenkamp and Kind 2013, Gutow et al. 2015, Arnold et al. 2015, Kim et al. 2019, Avila et al. 2020, Carral-Murrieta et al. 2023, Mendoza-Becerril et al. 2023, Carral-Murrieta et al. 2024). The macroalga *S.muticum* has the highest species richness (23 spp.; Withers et al. (1975), Norton and Benson (1983), Wernberg et al. (2004), Gutow et al. (2015), Carral-Murrieta et al. (2024)), followed by *A.spicifera* (14 spp.; Migotto (1996), Oliveira and Marques (2007), Oliveira and Marques (2011)) and *G.parvispora* (8 spp.; this study).

The hydrozoans were mainly found in the middle region of the *G.parvispora* thallus, in contrast to the benthic *Sargassum* species and *Cystoseiraamentacea* (C.Agardh) Bory, where the basal region hosted the highest number of hydrozoan species (Fraschetti et al. 2006, Carral-Murrieta et al. 2024). This contrast may be related to the preference for sandy substrates of *G.parvispora* as opposed to benthic *Sargassum* species and *C.amentacea* for rocky reefs, as well as the competition for the surrounding fauna and the growth of other algae. Moreover, colonial invertebrate larvae avoid settling in regions of the alga with members of competitively dominant species; therefore, the epibionts tend to settle on the youngest parts (Stebbing 1971, Grosberg 1981). For this reason, the epibiont fauna is usually smaller in the apical area because of the high growth rate and renewal of filaments in this zone in the algae of the genus *Gracilaria* (Molina-Vargas and Álvarez-León 2014). Furthermore, the richness and abundance of hydroids also depend on the macroalgal morphology. For instance, highly branched macroalgae have many micro-habitats that facilitate hydroid settlement and persistence in macroalgae such as *Cystoseirabarbata* (Stackhouse) C.Agardh, *C.amentacea* and *Sargassum* spp. (Faucci and Boero 2000, Carral-Murrieta et al. 2024), while *G.parvispora* has a single dominant axis, usually with three branching orders.

### Conclusions

This study demonstrated that the macroalga *G.parvispora* is a basibiont hosting colonial sessile epibionts, with the most frequent group being hydrozoans. This is the first time that the associated fauna of this macroalga has been studied and it provides essential information on the taxonomy and diversity of their epibionts. However, since macrophytes are potential vectors for species introductions in other regions (Kuhlenkamp and Kind 2013), further studies on non-native or invasive macroalgae and their epibionts are needed to assess whether these algae are conducive for the introduction of bryozoan or hydrozoan species into the local fauna. It is also important to assess whether the presence of colonial epibionts is directly related to their geographical distribution, whether they exhibit opportunistic settlement on the substrate (Oliveira and Marques 2011) or whether they prefer specific lineages of macrophytes for their development (Munari et al. 2015). Having established the basis for locating the macroalga in La Paz Bay and identifying its epibionts, it is possible to propose a standardised methodology for analysing whether the diversity and abundance of the epifauna depend on the macroalgae's morphology, as Gan et al. (2019) suggested for the epifauna associated with different macroalgae. The methodology of future studies should also consider the incorporation of the variation of environmental parameters.

## Supplementary Material

XML Treatment for Hydroidolina

XML Treatment for “Anthoathecata”

XML Treatment for "Filifera"

XML Treatment for Oceaniidae

XML Treatment for Corydendrium

XML Treatment for Corydendrium sp.

XML Treatment for Leptothecata

XML Treatment for Macrocolonia

XML Treatment for Plumupheniida

XML Treatment for Plumulariida

XML Treatment for Kirchenpaueriidae

XML Treatment for Ventromma

XML Treatment for Ventromma halecioides

XML Treatment for Plumulariidae

XML Treatment for Plumularia

XML Treatment for Plumularia floridana

XML Treatment for Statocysta

XML Treatment for Proboscoida

XML Treatment for Obeliida

XML Treatment for Clytiidae

XML Treatment for Clytia

XML Treatment for Clytia linearis

XML Treatment for Clytia sp.

XML Treatment for Obeliidae

XML Treatment for Obelia

XML Treatment for Obelia cf. dichotoma

XML Treatment for Obelia oxydentata

XML Treatment for Obelia tenuis

48ECB321-E7D5-534E-9AE3-37F7287DB22D 10.3897/BDJ.12.e130248.suppl1 Supplementary material 1 Epibionts on the introduced macroalga Gracilariaparvispora in La Paz Bay
Data type occurrences Brief description Epibionts on the introduced macroalga *Gracilariaparvispora* in La Paz Bay. File: oo_1115477.xlsx https://binary.pensoft.net/file/1115477 Mendoza-Becerril, M.A., Murillo-Torres, P., León-Cisneros, K.

## Figures and Tables

**Figure 7.**
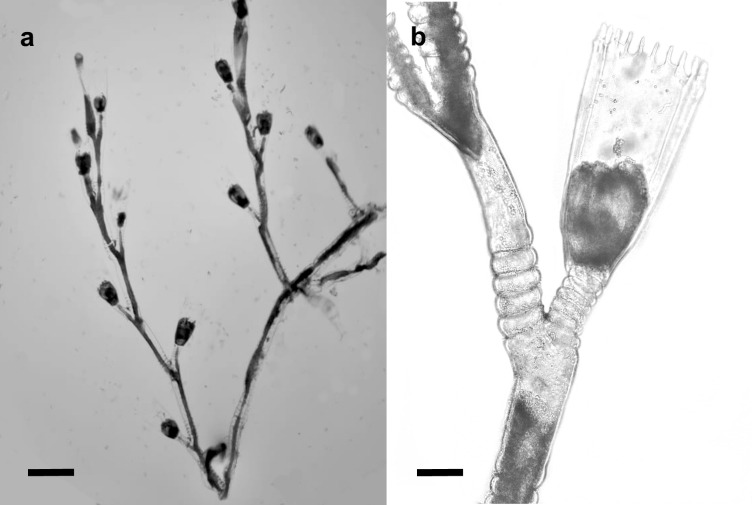
*Obeliaoxydentata*. **a** colony, scale equals 0.5 mm; **b** part of hydrocaulus with hydrothecae; scale equals 0.1 mm.

**Table 1.** Data of herbarium (H) and field (F) samples on *Gracilariaparvispora* from La Paz Bay, Baja California Sur. Latitude (N), Longitude (W), Temperature (°C), Salinity (PSU), Substrate (Sub.), Depth (m), No data (ND).

**Sites**	**N**	**W**	**Sample**	°**C**	**PSU**	**Sub.**	**m**	**Year**	**Month**
Port of San Juan de la Costa	24.398	110.681	F	27	31	rock and sand	0.5 – 7.0	2021	Nov.
ROFOMEX SJC	24.367	110.679	F	23	ND	sand	0.5 – 1.5	2021	May
El Mogote	ND	ND	H	ND	ND	sand	ND	2013	Jul.
La Concha	24.202	110.300	H	ND	ND	sand	0.5 – 1.5	2008	Apr.
	24.202	110.300	H	ND	ND	sand	0.5 – 1.5	2009	Mar.
	24.202	110.300	H	ND	ND	sand	0.5 – 1.5	2013	Jul.
	24.202	110.300	F	ND	ND	sand	0.5 – 1.5	2021	Jun.
	24.202	110.300	F	18	36	sand	0.5 – 1.5	2022	Feb.
El Caimancito	24.206	110.301	H	ND	ND	rock and sand	0.5 – 1.5	1980	Nov.
	24.206	110.301	H	ND	ND	rock and sand	0.5 – 1.5	2002	Mar.
UABCS Pichilingue	24.270	110.325	F	28	35	sand	0.5 – 3.0	2021	Jul.
	24.270	110.325	F	25	35	sand	0.5 – 3.0	2022	Feb.
Punta Diablo	24.316	110.340	F	25	33	rock and coral	0.5 – 8.0	2021	Jul.
	24.316	110.340	F	25	33	rock and coral	0.5 – 8.0	2022	Apr.
	24.316	110.340	F	23	35	rock and coral	0.5 – 8.0	2022	Jul.

**Table 2.** SIMPER (similarity percentage) analysis results demonstrated that taxa accounted for the most similarity within each month and the most dissimilarity between months.

**Within group**	**Average similarity**	**Contribution** %	**Cumulative contribution** %
**February**			
Obeliacf.dichotoma	16.2	79.77	79.77
*Ventrommahalecioides*	3.69	18.16	97.33
**May**			
Obeliacf.dichotoma	55.45	85.64	85.64
**August**			
*Clytialinearis*	8.89	39.72	39.72
Obeliacf.dichotoma	6.63	29.62	69.34
*Ventrommahalecioides*	6.13	27.38	96.72
**Between groups**			
**February and May**			
Obeliacf.dichotoma	32.86	50.85	50.85
*Ventrommahalecioides*	19.34	29.93	80.78
**February and August**			
Obeliacf.dichotoma	26.85	32.46	32.46
*Ventrommahalecioides*	20.45	24.73	57.19
*Clytialinearis*	18.81	22.75	79.94
**May and August**			
Obeliacf.dichotoma	29.85	41.82	41.82
*Ventrommahalecioides*	17.81	24.96	66.78
*Clytialinearis*	13.54	18.97	85.75
